# A perl package and an alignment tool for phylogenetic networks

**DOI:** 10.1186/1471-2105-9-175

**Published:** 2008-03-27

**Authors:** Gabriel Cardona, Francesc Rosselló, Gabriel Valiente

**Affiliations:** 1 Department of Mathematics and Computer Science, University of the Balearic Islands, E-07122 Palma de Mallorca, Spain; 2 Algorithms, Bioinformatics, Complexity and Formal Methods Research Group, Technical University of Catalonia, E-08034 Barcelona, Spain

## Abstract

**Background:**

Phylogenetic networks are a generalization of phylogenetic trees that allow for the representation of evolutionary events acting at the population level, like recombination between genes, hybridization between lineages, and lateral gene transfer. While most phylogenetics tools implement a wide range of algorithms on phylogenetic trees, there exist only a few applications to work with phylogenetic networks, none of which are open-source libraries, and they do not allow for the comparative analysis of phylogenetic networks by computing distances between them or aligning them.

**Results:**

In order to improve this situation, we have developed a Perl package that relies on the BioPerl bundle and implements many algorithms on phylogenetic networks. We have also developed a Java applet that makes use of the aforementioned Perl package and allows the user to make simple experiments with phylogenetic networks without having to develop a program or Perl script by him or herself.

**Conclusion:**

The Perl package is available as part of the BioPerl bundle, and can also be downloaded. A web-based application is also available (see availability and requirements). The Perl package includes full documentation of all its features.

## Background

**Figure 1:**
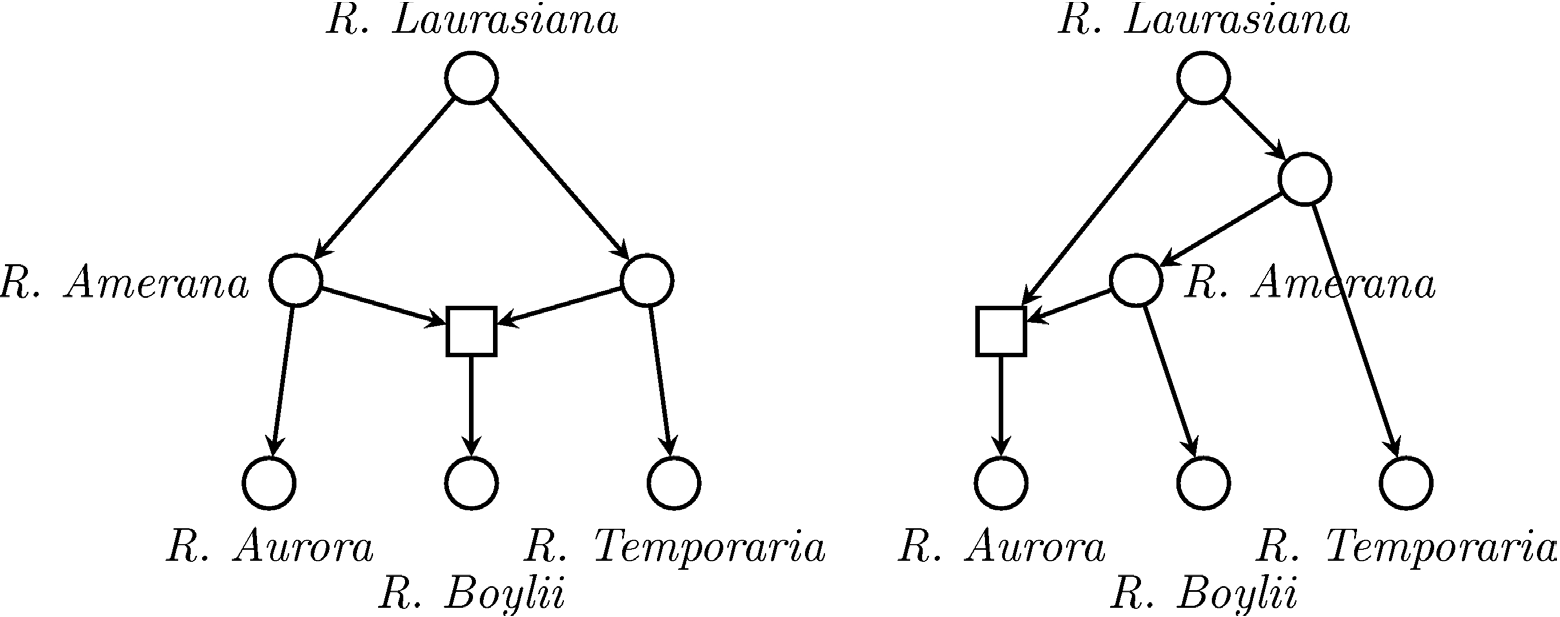
**A reticulation event in a phylogeny**. A hypothetical reticulation event between the *R. Amerana *and *R. Laurasiana *groups in two phylogenies inferred from evolutionary distances among three species of frog: *R. Aurora*, *R. Boylii *and *R. Temporaria *[3].

Phylogenetic networks have been studied over the last years as a richer model of the evolutionary history of sets of organisms than phylogenetic trees, because they take into account not only mutation events but also evolutionary events acting at the population level, like recombination between genes, hybridization between lineages, and lateral gene transfer. The latter turn phylogenies into reticulate networks, which are best modeled as directed acyclic graphs [1,2]. For instance, Figure 1 shows two phylogenies inferred from evolutionary distances among three species of frog: *R. Aurora*, *R. Boylii *and *R. Temporaria *[3], enriched with a hypothetical reticulation event (between the *R. Amerana *and *R. Laurasiana *groups), which turned them into phylogenetic networks.

We briefly recall below some definitions and results from [4] on phylogenetic networks. See [5] for an introduction to reticulation in phylogenetic analysis.

A *phylogenetic network *on a set *S *of taxa is any rooted directed acyclic graph whose leaves (those nodes without outgoing edges) are bijectively labeled by the set *S*.

Let *N *= (*V, E*) be a phylogenetic network on *S*. A node *u *∈ *V *is said to be a *tree node *if it has, at most, one incoming edge; otherwise it is called a *hybrid node*. A phylogenetic network on *S *is a *tree-child phylogenetic network *if every node either is a leaf or has at least one child that is a tree node. Tree-child phylogenetic network include galled-trees [6,7] as a particular case.

Let *S *= {ℓ_1_, ..., ℓ_*n*_} be the set of leaves. We define the *μ-vector *of a node *u *∈ *V *as the vector *μ*(*u*) = (*m*_1_(*u*), ..., *m*_*n*_(*u*)), where *m*_*i*_(*u*) is the number of different paths from *u *to the leaf ℓ_*i*_. The multiset *μ*(*N*) = {*μ*(*v*) | *v *∈ *V*} is called the *μ-representation *of *N *and, provided that *N *is a tree-child phylogenetic network, it turns out to completely characterize *N*, up to isomorphisms, among all tree-child phylogenetic networks on *S*.

This allows us to define a distance on the set of tree-child phylogenetic networks on *S*: the *μ-distance *between two given networks *N*_1 _and *N*_2 _is the symmetric difference of their *μ*-representations,

*d*_*μ*_(*N*_1_, *N*_2_) = |*μ*(*N*_1_) Δ *μ*(*N*_2_)|.

This defines a true distance, and when *N*_1 _and *N*_2 _are phylogenetic trees, it coincides with the well-known partition distance [8].

This representation also allows us to define an optimal alignment between two tree-child phylogenetic networks on *S*, say *n *= *|S|*. Given two such networks *N*_1 _= (*V*_1_, *E*_1_) and *N*_2 _= (*V*_2_, *E*_2_) (where, for the sake of simplicity, we assume *|V*_1_| ≤ |*V*_2_|), an *alignment *is just an injective mapping *M *: *V*_1 _→ *V*_2_. The *weight *of this alignment is

$$w(M)=\sum_{v\in V_{1}}\left(\left||\mu (v)-\mu (M(v))||+\chi (v,M(v)\right)\right),$$

where || · || stands for the Manhattan norm of a vector and *χ *(*u*, *v*) is 0 if both *u *and *v *are tree nodes or hybrid nodes, and 1/(2*n*) if one of them is a tree node and the other one is a hybrid node. An *optimal alignment *is, then, an alignment with minimal weight, which can be computed using the Hungarian algorithm [9].

## Implementation and results

### The extended Newick format

The eNewick (for "extended Newick") string defining a phylogenetic network appeared in the packages PhyloNet [10] and NetGen [11] related to phylogenetic networks, with some differences between them. The former encodes a phylogenetic network with *k *hybrid nodes as a series of *k *trees in Newick format, while the latter encodes it as a single tree in Newick format but with *k *repeated nodes.

Whereas the Perl module we introduce here accepts both formats as input, a complete standard for eNewick is implemented, based mainly on NetGen and following the suggestions of D. Huson and M. M. Morin (among others), to make it as complete as possible. The adopted standard has the practical advantage of encoding a whole phylogenetic network as a single string, and it also includes mandatory tags to distinguish among the various hybrid nodes in the network.

The procedure to obtain the eNewick string representing a phylogenetic network *N *goes as follows: Let {*H*_1_, ..., *H*_*m*_} be the set of hybrid nodes of *N*, ordered in any fixed way. For each hybrid node *H *= *H*_*i*_, say with parents *u*_1_, *u*_2_, ..., *u*_*k *_and children *v*_1_, *v*_2_, ..., *v*_ℓ_: split *H *in *k *different nodes; let the first copy be a child of *u*_1 _and have all *v*_1_, *v*_2_, ..., *v*_ℓ _as its children; let the other copies be children of *u*_2_, ..., *u*_*k *_(one for each) and have no children. Label each of the copies of *H *as

[label]# [type]tag [:branch_length]

where the parameters are:

• label (optional) string providing a labelling for the node;

• type (optional) string indicating if the node *H *corresponds to a hybridization (indicated by H) or a lateral gene transfer (indicated by LGT) event; note that other types can be considered in the future;

• tag (mandatory) integer *i *identifying the node *H *= *H*_*i*_.

• branch_length (optional) number giving the length of the branch from the copy of *H *under consideration to its parent.

We obtain a tree from this procedure whose set of leaves is the set of leaves of the original network together with the set of hybrid nodes (possibly repeated). The Newick string of the obtained tree (note that some internal nodes will be labeled and some leaves will be repeated) is the eNewick string of the phylogenetic network. The leftmost occurrence of each hybrid node in an eNewick string corresponds to the full description of the network rooted at that node. Although node labels are optional, all labeled occurrences of a hybrid node in an eNewick string must carry the same label.

**Figure 2:**
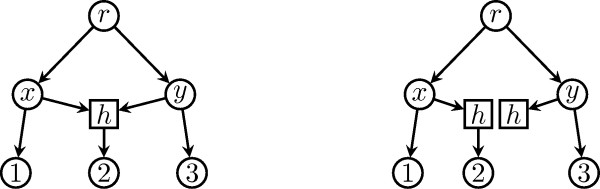
**Computing the eNewick string of a phylogenetic network**. A phylogenetic network *N *(left), and tree (right) associated to *N *for computing its eNewick string.

Consider, for example, the phylogenetic network depicted together with its decomposition in Figure 2. The eNewick string for this network would be ((1, (2)#H1), (#H1,3)); or ((1, (2)h#H1)x, (h#H1,3)y)r; if all internal nodes are labeled. The leftmost occurrence of the hybrid node in the latter string corresponds to the full description of the network rooted at that node: (2)h#H1.

The procedure to recover a network from its eNewick string simply requires recovering the tree and identifying those nodes that are labeled as hybrid nodes with the same identifier.

**Figure 3:**
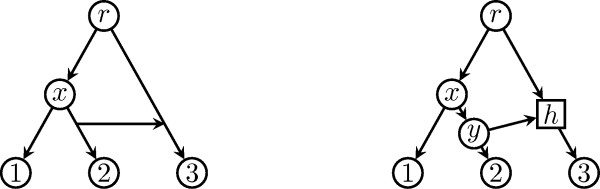
**Representing a lateral gene transfer event as a hybrid node**. Representation of a lateral gene transfer event (left) as a hybrid node in a phylogenetic network (right).

Notice that gene transfer events can be represented in a unique way as hybrid nodes. Consider, for example, the lateral gene transfer event depicted in Figure 3, where a gene is transferred from species 2 to species 3 after the divergence of species 1 from species 2. The eNewick string ((1, (2, (3)h#LGT1)y)x, h#LGT1)r; describes such a phylogenetic network. A program interpreting the eNewick string can use the information on node types in different ways; for instance, to render tree nodes circled, hybridization nodes boxed, and lateral gene transfer nodes as arrows between edges.

### The perl module

The Perl module Bio::PhyloNetwork, available as part of the BioPerl bundle [12], implements all the data structures needed to work with tree-child phylogenetic networks, as well as algorithms for:

• reconstructing a network from its eNewick string (in all its different flavours),

• reconstructing a network from its *μ*-representation,

• exploding a network into the set of its induced subtrees,

• computing the *μ*-representation of a network and the *μ*-distance between two networks,

• computing an optimal alignment between two networks,

• computing tripartitions [13,14] and the tripartition error between two networks, and

• testing if a network is time consistent [15], and in such a case, computing a temporal representation.

The underlying data structure is a Graph::Directed object, with some extra data, for instance the *μ*-representation of the network. It makes use of the Perl module Bio::PhyloNetwork::muVector that implements basic arithmetic operations on *μ*-vectors. Two extra modules, Bio::PhyloNetwork::Factory and Bio::PhyloNetwork::RandomFactory, are provided for the sequential and random generation (respectively) of all tree-child phylogenetic networks on a given set of taxa.

### The web interface and the java applet

The web interface allows the user to input one or two phylogenetic networks, given by their eNewick strings. A Perl script processes these strings and uses the Bio::PhyloNetwork package to compute all available data for them, including a plot of the networks that can be downloaded in PS format; these plots are generated through the application GraphViz and its companion Perl package.

Given two networks on the same set of leaves, their *μ*-distance is also computed, as well as an optimal alignment between them. The algorithm to compute such an alignment relies on the Hungarian algorithm [9]. If their sets of leaves are not the same, their *topological restriction *on the set of common leaves is first computed followed by the *μ*-distance and an optimal alignment.

A Java applet displays the networks side by side, and whenever a node is selected, the corresponding node in the other network (with respect to the optimal alignment) is highlighted, provided it exists. This is also extended to edges. Similarities between the networks are thus evident at a glance and, since the weight of each matched node is also shown, it is easy to see where the differences are.

## Conclusion

The Perl module Bio::PhyloNetwork relies on the BioPerl bundle and implements several algorithms on phylogenetic networks, from parsing and temporal representation to distances between phylogenetic networks and optimal alignments. The companion Java applet and web-based application make use of the Bio::PhyloNetwork module and allow the user to make simple experiments with phylogenetic networks without having to develop a program or Perl script by him or herself.

While the Bio::PhyloNetwork module computes distances between galled-trees and tree-child phylogenetic networks, it will also support the more general tree-sibling phylogenetic networks in a next release.

## Availability and requirements

The Perl package is available as part of the BioPerl bundle, at the url . It can also be downloaded from the url  (see Additional file 1). The web-based application is available at the url . The Perl package includes full documentation of all its features.

## Authors' contributions

All authors conceived the method, prepared the manuscript, contributed to the discussion, and have approved the final manuscript. GC implemented the software. GV also implemented part of the software.

## Supplementary Material

Additional file 1 **Bio-PhyloNetwork**. Compressed (gzip) archive (tar) of the perl module Bio::PhyloNetwork (containing the files Bio/PhyloNetwork/Factory.pm, Bio/PhyloNetwork/RandomFactory.pm, Bio/PhyloNetwork/muVector.pm, Bio/PhyloNetwork/FactoryX.pm, Bio/PhyloNetwork/TreeFactory.pm, Bio/PhyloNetwork/GraphViz.pm, Bio/PhyloNetwork/TreeFactoryMulti.pm, and Bio/PhyloNetwork/TreeFactoryX.pm) and the corresponding test module (containing the files Bio/PhyloNetwork/t/Factory.t, Bio/PhyloNetwork/t/TreeFactory.t, Bio/PhyloNetwork/t/muVector.t, Bio/PhyloNetwork/t/GraphViz.t, Bio/PhyloNetwork/t/RandomFactory.t, Bio/PhyloNetwork/t/lib/BioperlTest.pm, Bio/t/PhyloNetwork.t, and Bio/t/lib/BioperlTest.pm). Click here for file
